# Vesicles Secreted by Renal Cell Carcinoma Cells Cause Vascular Endothelial Cells to Express PSMA and Drive Tumor Progression

**DOI:** 10.3390/cells14030165

**Published:** 2025-01-22

**Authors:** Ryuta Watanabe, Keito Kagimoto, Mami Chosei, Tomohisa Sakaue, Mie Kurata, Noriyoshi Miura, Riko Kitazawa, Tadahiko Kikugawa, Shigeki Higashiyama, Takashi Saika

**Affiliations:** 1 Department of Urology, Ehime University Graduate School of Medicine, Toon 791-0295, Japan; i401025b@mails.cc.ehime-u.ac.jp (K.K.); miura.noriyoshi.mk@ehime-u.ac.jp (N.M.); takikuga@m.ehime-u.ac.jp (T.K.); saika.takashi.ol@ehime-u.ac.jp (T.S.); 2 Department of Biochemistry and Molecular Genetics, Ehime University Graduate School of Medicine, Toon 791-0295, Japan; ma-po@m.ehime-u.ac.jp (M.C.); shigeki@m.ehime-u.ac.jp (S.H.); 3 Department of Cardiovascular and Thoracic Surgery, Ehime University Graduate School of Medicine, Toon 791-0295, Japan; sakaue@m.ehime-u.ac.jp; 4 Division of Cell Growth and Tumor Regulation Proteo-Science Center, Ehime University, Toon 791-0295, Japan; 5 Department of Analytical Pathology, Ehime University Graduate School of Medicine, Toon 791-0295, Japan; miekrt@m.ehime-u.ac.jp; 6 Department of Pathology, Ehime University Graduate School of Medicine and Proteo-Science Center, Toon 791-0295, Japan; 7 Division of Diagnostic Pathology, Ehime University Hospital, Toon 791-0295, Japan; riko@m.ehime-u.ac.jp; 8 Department of Oncogenesis and Growth Regulation, Osaka International Cancer Institute, Chuo-ku, Osaka 541-8567, Japan

**Keywords:** tumor angiogenesis of renal cell carcinoma, prostate-specific membrane antigen, tumor endothelial cells, human umbilical vascular endothelial cells, tube formation, kidney cancer surgically resected specimen

## Abstract

Prostate-specific membrane antigen (PSMA) protein expression is induced during prostate cancer progression and metastasis. Recently, we reported that PSMA-positive vesicles released by prostate cancer cell lines enhanced vascular endothelial cell angiogenesis and that PSMA may be involved in tumor angiogenesis. Similarly, it is known that PSMA is upregulated in peritumoral vessels in renal cell carcinoma (RCC). In this study, we investigated the significance and molecular function of PSMA in RCC. PSMA immunohistochemical staining confirmed PSMA presence only in perinephric tumor vessels, and PSMA intensity was strongly correlated with recurrence rate and venous invasion. Spatial gene expression analysis revealed that FOLH1 expression, which codes PSMA, was upregulated in tumor blood vessels around renal cancer, and that angiogenesis-related pathways were enhanced. The 10,000 g pellet fraction of the renal cancer cell lines Caki1- and ACHN-conditioned medium (CM) induced PSMA positivity in human umbilical vein endothelial cells (HUVECs) and enhanced tube formation. Mass spectrometry indicated that the 10,000 g pellet fraction contained various kinds of growth factors, like GDF15 and MYDGF. RNA sequencing showed that supplementing HUVECs with RCC cell CM-enhanced angiogenesis-related signaling pathways. Conclusively, microvesicle components secreted by RCC cells transform vascular endothelial cells into PSMA-positive cells, enhancing angiogenesis.

## 1. Introduction

Renal cell carcinoma (RCC) represents 3–5% of all cancers globally and has a generally poor prognosis [1,2]. The disease is characterized by abnormal angiogenesis, making it resistant to chemotherapy and radiotherapy, owing to its ability to evade the immune system and promote hypoxia. Therefore, targeting angiogenesis is an important therapeutic approach. Mutations in the von Hippel–Lindau tumor suppressor (*VHL*) gene are highly frequent in clear cell RCC (ccRCC), causing the overexpression of hypoxia-inducible factor (HIF). This effect, in turn, increases vascular endothelial growth factor A (VEGF-A) and platelet-derived growth factor (PDGF) levels, thereby facilitating tumor growth [3,4]. Since 2005, VEGF pathway-targeted tyrosine kinase inhibitors (TKIs) have largely replaced interleukin (IL)-2 and interferon (IFN)-α in treatment regimens [5,6]. Recently, new immunotherapeutic options, specifically immune checkpoint inhibitors (ICIs) targeting programmed cell death protein (PD)-1 or cytotoxic T-lymphocyte-associated protein (CTLA)-4, have been introduced [7,8]. The link between angiogenesis and anti-tumor immunity suggests that the combination of ICIs (anti-PD-1) with anti-angiogenic TKIs is a promising anti-cancer treatment [9]. Therefore, a deeper understanding of the mechanisms underlying tumor angiogenesis in RCC could pave the way for novel therapeutic agents to manage tumor progression.

Recently, the prostate-specific membrane antigen (PSMA) has been reported to be expressed on the endothelial cells of various solid tumors, including renal cancer, and to enhance angiogenic activity [10,11,12,13,14]. Initially, the underlying molecular function of the PSMA protein was thought to be as a type II transmembrane protein with both glutamate carboxypeptidase II (GCPII) and folate hydrolytic enzyme (FOLH) activities [15,16,17]. PSMA is highly expressed in prostate cancer, especially in intermediate-risk and high-risk cases with lymph node metastasis or distant metastasis. It is a reliable target for detecting metastatic lesions [18,19,20].

Notably, 68Ga-PSMA PET/CT-targeting PSMA has been established as an effective diagnostic tool for prostate cancer [21]. Lutetium-177-labeled anti-PSMA antibodies and PSMA ligands have been developed and applied clinically [22,23]. In contrast, although the PSMA expression levels are low in normal human endothelial cells, in endothelial cells in various solid tumor tissues (e.g., thyroid, glioma, breast, non-small cell lung, colon, and renal cell carcinomas) they are high. In addition, tumor angiogenic activity is significantly reduced in mice lacking PSMA [16]. Watanabe et al. [24] recently demonstrated that PSMA-positive vesicles released by prostate cancer cells promoted angiogenesis by increasing the activity of endothelial cells. While it was previously believed that PSMA was not expressed in blood vessels surrounding prostate tumors, that study showed otherwise. Using fluorescence double staining, it was confirmed that PSMA was highly expressed in the tumor vasculature around prostate cancer. Additionally, tube formation assays indicated that microvesicles in a conditioned medium could convert endothelial cells to PSMA-positive cells, further enhancing angiogenesis. Previous studies have also demonstrated that PSMA promotes angiogenesis in gliomas [25]. In a study using glioma tissues from 60 patients who underwent surgery, immunohistochemical staining revealed high PSMA expression in the endothelial cells of glioblastoma blood vessels, and this expression was shown to enhance angiogenesis. It was also reported that PSMA interacted with integrin beta-4, leading to the activation of the NF-κB signaling pathway. In a xenograft mouse model using U87-luc cells, the administration of the PSMA inhibitor 2-PMPA after tumor formation resulted in significant tumor growth suppression. These findings suggest that PSMA plays a crucial role in glioblastoma progression, and may be a promising therapeutic target in glioma treatment [25].

Recently, the utility of PSMA PET CT in RCC has been investigated, including clinical studies highlighting its potential in staging RCC and detecting metastatic sites compared to computed tomography (CT) or magnetic resonance imaging (MRI) [26,27,28,29,30]. However, the significance of PSMA expression in the peritumoral vessels and the mechanism underlying PSMA positivity in RCC is unclear. In this study, we focused on RCC and evaluated the correlation between PSMA expression in peritumoral vessels and prognosis in surgically resected renal cancer lesions. In addition, we investigated the molecular function of PSMA in tumor angiogenesis in RCC cell lines.

## 2. Materials and Methods

### 2.1. Antibodies

The following antibodies were purchased from the indicated manufacturers (Table 1). 

### 2.2. Cell Culture

Lymph node carcinoma of the prostate (LNCaP), Caki1, and ACHN cells were purchased from the American Type Culture Collection (ATCC) (Manassas, VA, USA) and maintained at 37 °C with 5% CO_2_ in Roswell Park Memorial Institute (RPMI), Eagle’s minimum essential medium (EMEM), and McCoy’s 5A (all from Wako, Osaka, Japan), respectively, supplemented with 10% fetal bovine serum (FBS), 20 U/mL penicillin, and 100 µg/mL streptomycin. HEK293T cells were maintained at 37 °C in an atmosphere of 5% CO_2_ in Dulbecco’s minimum essential medium (DMEM, Wako, Osaka, Japan) supplemented with 10% FBS, 20 U/mL penicillin, and 100 µg/mL streptomycin. 

Human umbilical vein endothelial cells (HUVECs) were purchased from Lonza (Tokyo, Japan) and were maintained at 37 °C in an atmosphere of 5% CO_2_ in an endothelial basal medium (EBM)-2 (Lonza, Tokyo, Japan) according to the manufacturer’s instructions. HUVECs at passages 2–4 were used for experiments. HUVECs cultured on collagen I gels were treated with a conditioned medium (CM).

### 2.3. Transfection

Transfection was performed as previously described [14]. GeneJuice (Millipore, Burlington, MA, USA) was used for transfecting plasmids into HEK293T cells, according to the manufacturer’s instructions. The cells were used for subsequent experiments 48 h post transfection. Renal cell carcinoma cells were transfected with small interfering RNAs (siRNAs, 10 nM) using RNAimax (Invitrogen, Waltham, MA, USA) according to the manufacturer’s instructions. Subsequent experiments were performed at 72 h post-transfection. The following validated siRNA duplex oligomers were purchased and used for knockdown experiments: GGGCGAUCUAGUGUAUGUUAACUAU (PSMA siRNA, Invitrogen). The control siRNA was purchased from Sigma-Aldrich (SIC-001; St. Louis, MO, USA).

### 2.4. Western Blotting

Western blotting was performed as previously described in [31]. In summary, proteins were analyzed by SDS-PAGE, transferred to PVDF membranes, and blocked with 5% skim milk in 0.05% Tween-20/TBS. Membranes were incubated with 1:1000 primary and 1:2000 peroxidase-conjugated secondary antibodies, and proteins were visualized via enhanced chemiluminescence (LAS-4000, GE Healthcare, Chicago, IL, USA). 

### 2.5. Reverse Transcription Polymerase Chain Reaction (RT-PCR)

Reverse transcription polymerase chain reaction (RT-PCR) was performed as previously described in [32]. The following pairs of primers were used: 5′-CAGCTGGAAATATCCTAAATCTGA-3′ (PSMA sense primer), 5′-TTGGATGAACAGGAATACTTGGAA-3′ (PSMA antisense primer), 5′-TGCACCACCAACTGCTTAGC-3′ (GAPDH sense primer), and 5′-GGCATGGACTGTGGTCATGAG-3′ (GAPDH antisense primer).

### 2.6. Immunofluorescence Staining

Immunofluorescence was performed as previously described [14]. Cells were fixed with 4% paraformaldehyde (PFA) in phosphate-buffered saline (PBS) for 30 min at 20 °C and permeabilized with 0.1% Triton X-100 in PBS for 15 min at room temperature. To stain endogenous PSMA, the cells were fixed with 10% trichloroacetic acid (TCA) in PBS for 15 min at 4 °C and permeabilized with 0.05% saponin in PBS for 5 min at room temperature. After blocking with 3% bovine serum albumin in PBS for 30 min at room temperature, the cells were incubated with 1:1000 primary antibodies and 1:2000 fluorophore-conjugated secondary antibodies. To stain the nuclei, the fixed cells were treated with Hoechst 33342 (1:2000; Molecular Probes, Eugene, OR, USA) at room temperature for 1 h.

### 2.7. Confocal Microscopy

Fluorescence images were obtained using an A1R laser confocal microscope (Nikon, Tokyo, Japan) equipped with a 60 × 1.27 Plan-Apochromat water immersion lens. Images were analyzed using ImageJ (Version 1.54m 5 December 2024) or FIJI software (Version 2.16.0) (National Institutes for Health [NIH], Bethesda, MD, USA).

### 2.8. Preparation of CM

To test our hypothesis that PSMA promotes tumorigenesis by transforming endothelial cells from PSMA-negative to -positive tumor endothelial cells via a mechanism similar to that observed in prostate cancer, we generated conditioned media (CM) from several human RCC (Caki1 and ACHN) and prostate (LNCaP) cancer cell lines. CM was prepared as previously described [14].

Briefly, prostate cancer cells (1.5 × 10^5^ cells/mL) were seeded in six-well plates, with each well coated with 500 µL collagen I gel. Then, 3 days later, the culture medium in each well was replaced with 2 mL fresh RPMI medium, followed by incubation for 3 days, and then the medium was collected and centrifuged at 1000× *g* for 5 min. The collected supernatant was designated as CM. To determine the concentration of a 10,000× *g* pellet fraction of CM, 10 mL was centrifuged at 10,000× *g*, the supernatants were removed, and then the pellet was resuspended in 1 mL EBM-2.

### 2.9. Tube Formation Assay on Collagen I Gels

Tube formation assays were performed as described previously in [33], and tube lengths were measured using ImageJ or FIJI software.

### 2.10. Centrifugation

Next, Caki1 and ACHN cell-derived CMs were divided into 10,000 and 100,000× *g* pellets, while the supernatant was fractionated using centrifugation into the 100,000× *g* fraction. Half of the 100,000× *g* supernatant was filtered to remove large microsomes and HUVECs were then cultured in the CM fractions derived from LNCaP cells. The CM was centrifuged at 1000× *g* for 10 min at 4 °C to obtain the pellet debris. The supernatant medium samples were centrifuged at 10,000× *g* for 10 min at 4 °C, and the pellets were designated as 10,000× *g* pellets. The supernatant was transferred to an ultracentrifuge tube for further centrifugation at 100,000× *g* for 60 min at 4 °C (Optima XL80K, Beckman Coulter Inc., Indianapolis, IN, USA), and the pellets were designated as 100,000× *g* pellets. The collected supernatant was passed through a 0.35 µm filter to remove the larger protein aggregates or vesicles.

### 2.11. Immunohistochemistry

The 10% neutral buffered formalin-fixed and paraffin-embedded (FFPE) tissue samples were cut into 3–5 µm thick sections using a microtome and stained according to the standard protocols for immunohistochemical evaluation. The antibodies used were mouse monoclonal anti-PSMA (M3620, Dako, Glostrup, Denmark) and rabbit polyclonal anti-CD31 (ab28364, Abcam, Cambridge, UK) antibodies. Human prostate tumor samples were prepared from surgical samples fixed in 10% neutral-buffered formalin. All samples were evaluated by pathologists at Ehime University. Sampling and methods for histological investigation were approved by the local institutional review board and ethics committee of Ehime University Hospital (approval no. 1812008).

### 2.12. Spatial Transcriptomics (Visium HD)

Spatial transcriptomic analyses were performed as previously described in [34,35]. FFPE samples that passed RNA quality control (DV200 > 30%) were used for spatial transcriptomic construction and sequencing. Tissues were prepared according to the Visium HD Spatial Gene Expression for FFPE tissue preparation guide (CG000518, 10x Genomics, Pleasanton, CA, USA). The resected kidney tissue specimens were thinly sliced (10 µm thick), and Visium HD was performed on a designated area of 6.5 × 6.5 mm^2^, including the peripheral tumor and normal vessels sites. The tissue was permeabilized and the mRNA was reverse-transcribed into cDNA using a barcode containing slide location information. 

cDNA was amplified and sequenced to generate spatially resolved gene expression data. One type of barcode was assigned to each spot to enable mRNA analysis while preserving positional information. The sequencing was performed at the Center for Gene Research at Yamaguchi University. Libraries were sequenced using MGI DNBSEQ-G400RS (MGI Tech Co., Shenzhen, China). The Space Ranger pipeline v2022.0705.1 (10x Genomics, CA, USA) and the GRCh38-2020-A reference were used to process FASTQ files.

The sequencing results were guaranteed to be accurate, as follows: number of 8 µm binned squares under tissue: 671,854; mean reads per 8 µm bin: 756.2; mean UMIs per 8 µm bin: 120.5; total genes detected: 18,008.

### 2.13. Statistical Analysis

Statistical comparisons were performed using a two-tailed Student’s *t*-test or one-way analysis of variance (ANOVA), followed by Tukey’s post hoc test. The log-rank test was used to test for significant differences in recurrence rates between the three groups in the Kaplan–Meier curve.

## 3. Results

### 3.1. Spatial Gene Expression Analysis Revealed That FOLH1 (PSMA) Expression Was Upregulated in Tumor Blood Vessels Around Renal Cancer and That Angiogenesis-Related Pathways Were Enhanced

**Figure 1:**
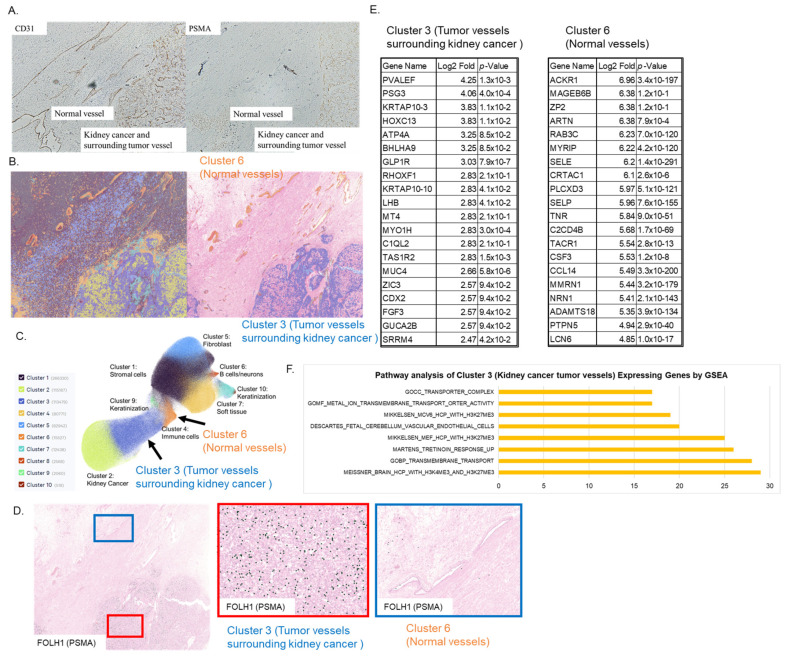
Spatial gene expression analysis revealed that FOLH1 (PSMA) expression was upregulated in tumor blood vessels around renal cancer and that angiogenesis-related pathways were enhanced. (**A**) Immunohistochemical staining revealed that compared with the vascular marker CD31 staining observed in adjacent sections, PSMA was expressed exclusively in the peritumoral vessels. (**B**) Spatial gene expression analysis (VisiumHD) categorized the kidney cancer tissue cells into 10 clusters, with cluster 10 identified as an independent cluster corresponding to the kidney cancer tumor vessels (Cluster 3) and normal vessels (Cluster 6), respectively. (**C**) In UMAP, Cluster 3 and Cluster 6 were recognized as adjacent clusters and thought to have a close genetic profile, but they were recognized as separate clusters. (**D**) FOLH1, which encodes PSMA, is highly expressed in clusters of tumor blood vessels (Cluster 3) around renal cancer but is hardly expressed in clusters of normal blood vessels (Cluster 6). (**E**) The 20 most highly expressed genes in kidney cancer tumor vessels (Cluster 3) and normal vessels (Cluster 6) are shown in (**F**). Pathway analysis of Cluster 3 by GSEA showed that there was an increase in multiple pathways involved in angiogenesis.

Immunohistochemical staining revealed that, compared with the vascular marker CD31 observed via staining in adjacent sections, PSMA was expressed exclusively in the peritumoral vessels (Figure 1A). Spatial gene expression analysis (Visium HD) categorized the kidney cancer tissue cells into 10 clusters, with cluster 10 identified as an independent cluster corresponding to the kidney cancer tumor vessels (Cluster 3) and normal vessels (Cluster 6), respectively. (Figure 1B,C). In UMAP, Cluster 3 and Cluster 6 were recognized as adjacent clusters and thought to have a close genetic profile, but they were recognized as separate clusters. FOLH1, which encodes PSMA, was highly expressed in clusters of tumor blood vessels (Cluster 3) around renal cancer, but was hardly expressed in clusters of normal blood vessels (Cluster 6) (Figure 1D).

The 20 most highly expressed genes in kidney cancer tumor vessels (Cluster 3) and normal vessels (Cluster 6) are shown in Figure 1E. Pathway analysis of Cluster 3 by GSEA showed that there was an increase in multiple pathways involved in angiogenesis. Specifically, it is thought that the increase in dimethylation at K4 (H3K4me2) and trimethylation at K27 (H3K27me3) of histone H3 induces angiogenesis-inducing transcription. In addition, there was an increase in the group of genes induced by tretinoin, which promotes angiogenesis (Figure 1F).

### 3.2. Intensity of PSMA Expression in Surrounding Tumor Vessels of RCC Correlated with Clinical Recurrence Rate

**Figure 2:**
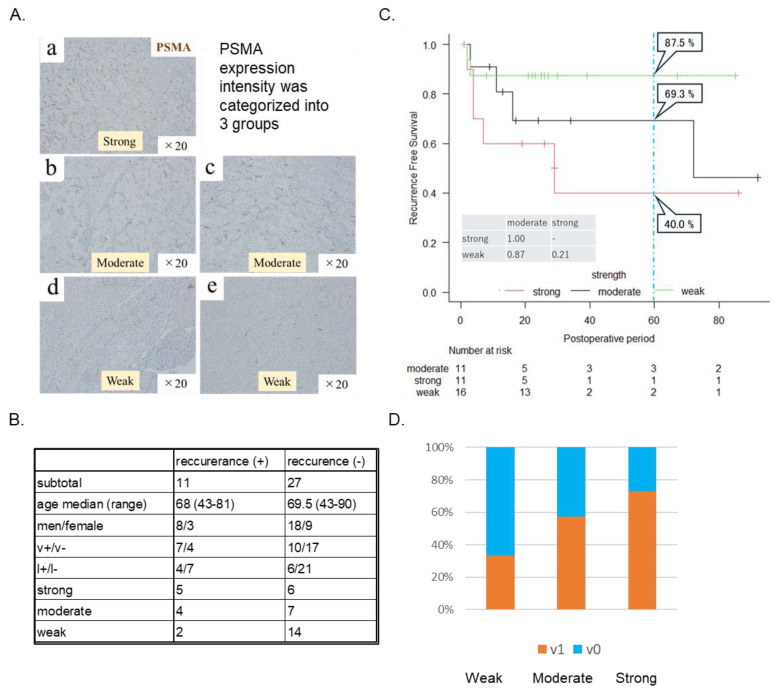
The intensity of prostate-specific membrane antigen (PSMA) expression in surrounding tumor vessels of renal cell carcinoma (RCC) is correlated with the clinical recurrence rate. (**A**) PSMA expression intensity was classified into three levels, namely strong, moderate, and weak, determined by the threshold using Image J (>70, 30–70, and <30, respectively). PSMA weak, moderate, and strong expression intensity was identified in 18, 11, and 16 cases, respectively. (**B**) Thirty-eight of forty-five patients were examined in follow-up visits. No significant differences were observed in patient background in the recurrence and non-recurrence groups. (**C**) The Kaplan–Meier curve of clinical recurrence rate generated for each PSMA expression intensity showed that the strong expression intensity group had predominantly higher recurrence rate than moderate and weak groups. (**D**) The strong group had an increased percentage of venous invasion, histologically.

PSMA protein expression was evaluated immunohistochemically in excised tissues from 45 patients with RCC who underwent total or partial surgical resection at Ehime University Hospital. We classified PSMA expression intensity at the following three levels, determined by the Image J intensity threshold: strong (>70), moderate (30–70), and weak (<30), with 16, 11, and 18 identified cases, respectively (Figure 2A). Furthermore, 38 of the 45 patients with follow-up visits were examined. 

Recurrence was defined as the point at which renal cancer developed local recurrence or distant metastasis after surgery. There were no significant differences in patient backgrounds in the recurrence and non-recurrence groups (Figure 2B). A Kaplan–Meier curve of the clinical recurrence rate was generated for each PSMA expression intensity. Recurrence was defined as a progressive disease (PD) on imaging or a change in treatment. The results showed that the strong expression group had a higher recurrence rate than the moderate and weak expression groups (Figure 2C). In addition, the strong group showed an increased percentage of venous invasion (Figure 2D). 

### 3.3. CM Derived from Caki1 and ACHN RCC Cell Lines Induced PSMA Expression in HUVECs

**Figure 3:**
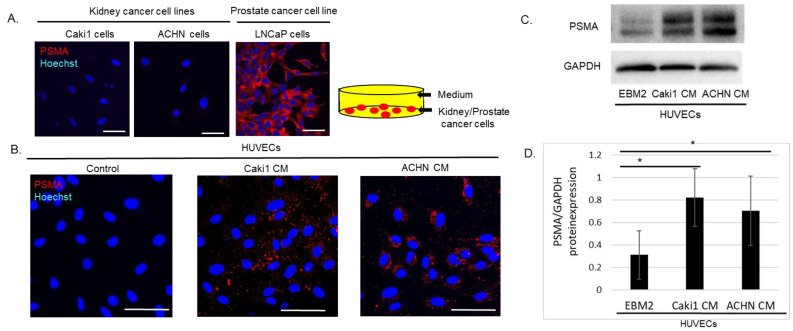
Conditioned medium (CM) derived from renal cell carcinoma (RCC) Caki1 and ACHN cells induced prostate-specific membrane antigen (PSMA) expression in human umbilical vein endothelial cells (HUVECs). (**A**) Conditioned medium (CM) was prepared from human RCC Caki1 and ACHN and prostate (LNCaP) cancer cell lines cultured on plastic dishes. LNCaP cells expressed PSMA, whereas the Caki1, and ACHN cell lines both did not express PSMA protein in tumor cells. Scale bars: 100 µm. (**B**) HUVECs seeded on collagen I gels were cultured with CM described in (**A**). CM from RCC cell lines was used. In contrast to CM derived from LNCaP cells, PSMA protein was also detected in HUVECs cultured in CM derived from Caki1 or ACHN cells. Scale bars: 100 µm. (**C**) Wb revealed that CM derived from Caki1 or ACHN cells, as well as CM derived from LNCaP cells, induced PSMA protein expression in HUVECs. (**D**) Quantitation of C. ratio of PSMA/GAPDH from three independent experiments was analyzed. Data show the mean ± SEM., * *p* < 0.05.

Tumor endothelial cells adjacent to renal carcinomas express PSMA, and the intensity correlates with the recurrence rate. Next, we prepared CM from human RCC (Caki1 and ACHN), and prostate (LNCaP) cells cultured on plastic dishes (Figure 3A). LNCaP cells expressed PSMA, whereas the Caki1 and ACHN RCC cells did not express PSMA in the tumors (Figure 3A). 

The results illustrated in Figure 3B show that HUVECs cultured in CM from LNCaP cells expressed PSMA protein, whereas those cultured in EBM-2 medium as a control expressed PSMA protein below the detection limit. Similarly to HUVECs cultured in CM derived from LNCaP cells, the PSMA protein was also detected in those cultured in CM derived from Caki1 or ACHN cells (Figure 3C). Treatment with CM derived from Caki1 or ACHN cells significantly increased PSMA protein expression in HUVECs (Figure 3D). In this study, the RCC cell lines cultured on plastic dishes and collagen I gels generated CM that induced PSMA expression in HUVECs (Figure 3A,B).

### 3.4. The 10,000× g Pellet Fraction of CM Derived from Caki1 and ACHN Cells Transformed HUVECs from PSMA-Negative to -Positive

**Figure 4:**
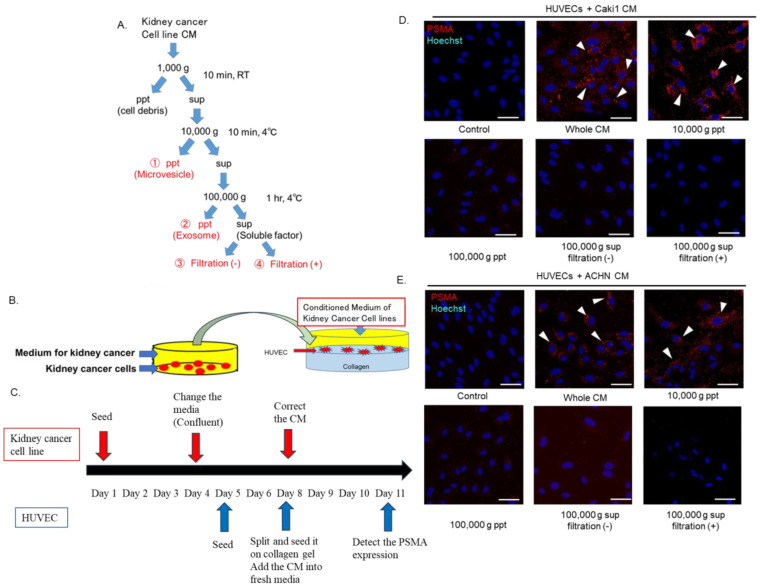
The 10,000× *g* pellet fractions of conditioned medium (CM) derived from Caki1 and ACHN cells transform human umbilical vein endothelial cells (HUVECs) from PSMA-negative to -positive. (**A**) Caki1 and ACHN cell-derived CMs were divided into 10,000 and 100,000× *g* pellets and 100,000× *g* supernatant. The supernatant was centrifuged to obtain the 100,000× *g* fraction and half of the 100,000× g supernatant was filtered to remove large microsomes. (**B**) HUVECs were cultured in CM fractions derived from LNCaP cells. (**C**) A flowchart of the study protocol. Confocal images of HUVECs cultured with each CM fraction derived from (**D**) Caki1 and (**E**) ACHN cells. Immunofluorescence staining for PSMA in HUVECs 72 h after they were seeded on collagen I gels and exposed to CM fractions diluted (1:1) with or suspended in endothelial basal medium (EBM)-2. PSMA-positive HUVECs are shown by arrowheads. Scale bars: 100 µm. Representative images from three independent experiments are shown.

To identify HUVECs that had been transformed from PSMA-negative to PSMA-positive, CM fractions derived from Caki1 and ACHN cells that had been cultured for 72 h in a confluent state were separated into a pellet at 10,000× *g* and a supernatant at 100,000× *g* (Figure 4A). HUVECs cultured in collagen gel were cultured in a medium containing a 1:1 mixture of CM fraction derived from renal cancer cells and fresh media (Figure 4B). After 72 h, PSMA expression in HUVECs was evaluated via Western blotting and immunostaining (Figure 4C). As shown in Figure 4D,E, HUVECs cultured in the 10,000 *g* pellet fraction expressed PSMA in the cytoplasm. HUVECs did not convert from PSMA-negative to PSMA-positive in other fractions (Figure 4D,E).

### 3.5. The 10,000× g Pellet Fraction of CM Derived from Caki1 and ACHN RCC Cells Promoted Angiogenesis In Vitro

**Figure 5:**
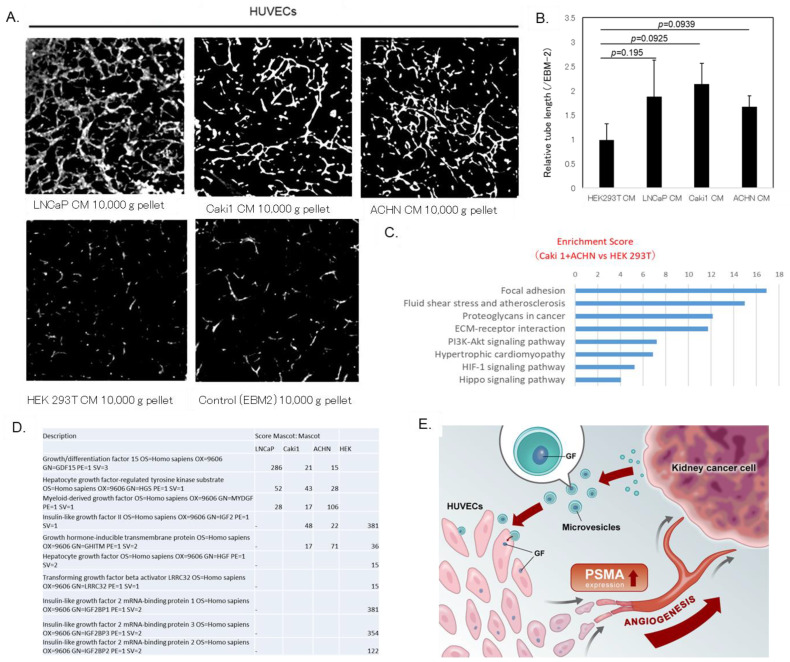
Tube formation assay of human umbilical vein endothelial cells (HUVECs) cultured with 10,000× *g* pellet fraction of conditioned medium (CM) derived from renal cell carcinoma (RCC) cells. (**A**) Representative images of tube formation. HUVECs seeded on collagen I gel were treated with 10,000× *g* pellet fraction of CM derived from Caki1 and ACHN cells for 6 h, and packed on collagen I followed by vascular endothelial growth factor (VEGF)-A stimulation for 66 h. HUVECs were stained with calcein-AM before acquiring images. (**B**) Total tube lengths from three independent experiments were measured and normalized to those of cells cultured with normal endothelial basal medium (EBM)-2. Data are means ± standard error of the mean (SEM). (**C**) CM samples from HEK293 (normal kidney), Caki1, and ACHN (RCC) cells were added to HUVECs, and gene expression changes were analyzed using RNA sequencing (RNA-seq). Pathway analysis showed that HUVECs treated with RCC cell culture supernatants enhanced the expression of angiogenesis-related signaling pathways such as the extracellular matrix (ECM), phosphoinositide 3-kinase (PI3K), AKT serine/threonine kinase (AKT), Hippo, and hypoxia-inducible factor (HIF)-1. (**D**) Mass spectrometry analysis of 10,000× *g* pellet fraction of RCC cell culture supernatants revealed factors such as growth differentiation factor 15 (GDF15), hepatocyte growth factor-regulated tyrosine kinase substrate (HGS), and myeloid-derived growth factor (MYDGF). (**E**) Microvesicle components secreted by renal cancer cells transport prostate-specific membrane antigen (PSMA) to vascular endothelial cells and enhance angiogenic activity.

The results of the tube formation assay of HUVECs mimicking angiogenesis to examine the effects of the 10,000× *g* pellet fraction from PSMA-expressing RCC cells on endothelial function showed that it promoted HUVEC tube formation (Figure 5A,B). In addition, the prostate cancer cell line showed similar tube formation-promoting effects. CM samples from the HEK293 (normal kidney), Caki1, and ACHN (RCC) cells were added to HUVECs, and gene expression changes were analyzed using RNA-seq. The results showed that HUVEC-supplemented culture supernatants of the RCC cell lines enhanced the expression of angiogenesis-related signaling pathways molecules, such as the extracellular matrix (ECM), phosphoinositide 3-kinase (PI3K)-AKT serine/threonine kinase (AKT), Hippo, and hypoxia-inducible factor (HIF)-1 (Figure 5C).

In addition, mass spectrometry analysis of the 10,000× *g* pellet fraction of the RRC cell line culture supernatants showed the presence of growth factors, such as growth differentiation factor 15 (GDF15), hepatocyte growth factor-regulated tyrosine kinase substrate (HGS), and myeloid-derived growth factor (MYDGF, Figure 5D).

## 4. Discussion

In this study, we investigated the significance and molecular function of PSMA in RCC. We found that a 10,000× *g* pellet fraction of the CM derived from RCC and LNCaP cells transformed endothelial cells from PSMA-negative to -positive. We also found that PSMA-positive but not -negative HUVECs promoted tube formation. Furthermore, immunohistochemical evaluation revealed that tumor endothelial cells adjacent to RCC cells in the resected specimens expressed PSMA. Moreover, we hypothesized that some RCC cell-derived factors mediated not only the transformation of HUVECs from PSMA-negative to -positive cells, but also induced PSMA mRNA expression in HUVECs. The data obtained from these experiments suggest that RCC cells can transform surrounding PSMA-negative vascular endothelial cells into PSMA-positive cells (Figure 5E). 

PSMA is well known to be highly expressed in prostate cancer tissue [18,19,20], but its expression in numerous other solid tumor tissues, including RCC, is not as well-documented. PSMA is also expressed in several tissues under normal conditions, including salivary glands, kidneys, epididymis, ovary, ileocecoids, and astrocytes. It is noteworthy that PSMA is not expressed in most tumor cells, but rather in the surrounding vascular endothelial cells [36,37]. Although PSMA is expressed in prostate cancer tumor cells, the results of the current study indicate that it is not expressed in RCC tumor cells, but is localized to vascular endothelial cells. PSMA expression in the surrounding tumor vasculature is also enhanced in recurrent high-grade RCCs. Although there was no significant difference between the three groups in a log-rank test of the Kaplan–Meier curve, this may be because the number of cases in the recurrent group was small. However, recurrence rates at 5 years were 87.5% in the strong group, 69.3% in the moderate group, and 40.0% in the weak group, and the recurrence rate was clearly high in the strong group. Previous studies consistent with our findings have reported that PSMA expression is predominantly localized to vascular endothelial cells, and is rarely observed in tumor cells in RCC [38]. These studies demonstrated that higher PSMA expression in tumor vasculature is associated with higher grade, advanced stage, and poor prognosis, including metastatic and lethal RCC cases. Notably, TCGA-based analyses confirmed that expression of FOLH1 mRNA—which encodes PSMA—in tumor-associated blood vessels holds prognostic significance at the transcriptional level, supporting the potential use of PSMA-targeted imaging in RCC [38].

These findings align with our observation of increased PSMA expression in the vasculature surrounding recurrent, high-grade RCC tumors. Unlike prostate cancer, where tumor cells themselves express PSMA, making it difficult to assess vascular expression, RCC tumor cells are PSMA-negative. This distinction allows for a unique opportunity to evaluate PSMA expression in the vasculature as a prognostic marker by analyzing vascular PSMA intensity in RCC. The results of the current study suggest that PSMA expression in the peritumoral vasculature of RCC may have clinical utility with respect to predicting the efficacy of anti-angiogenic therapies and patient prognoses. Given the success of PSMA-targeted therapies in prostate cancer, PSMA-targeting agents could be investigated as a potential therapeutic approach in RCC, particularly in ccRCC, where PSMA expression is associated with tumor aggressiveness. Recent studies further support this, showing that FOLH1 (PSMA-encoding gene) expression is associated with tumor angiogenesis and predicts progression-free survival in metastatic ccRCC (m-ccRCC) patients treated with VEGF inhibitors (VEGFi) [39]. These findings suggest that PSMA PET imaging could serve as a noninvasive diagnostic modality to guide CST selection (IO/IO vs. IO/VEGFi) and predict responses to VEGFi treatments in m-ccRCC [39]. While evidence for PSMA-targeted therapy in RCC remains limited, vascular PSMA expression shows promise as a predictive biomarker and warrants further investigation in metastatic settings and in combination with existing RCC therapies.

In this study, we introduced VisiumHD, the latest high-resolution spatial gene analysis, and were able to analyze the differences in gene expression between tumor blood vessels and normal blood vessels in renal cancer. Spatial gene analysis is an innovative technique that allows gene expression to be mapped on tissue slides while retaining positional information. In particular, VisiumHD has an extremely small spot diameter of 2 μm, and it is possible to visualize gene expression at almost the single-cell level. This helps in elucidating the mechanisms of tumor progression by focusing on each cell in the tumor microenvironment. In this study, we applied this technology to determine that the expression of FOLH1, which encodes PSMA, is upregulated in tumor blood vessels surrounding renal cancer, and that the angiogenesis-related pathway is also upregulated in these areas. In particular, it has been reported that increases in the dimethylation (H3K4me2) and trimethylation (H3K27me3) of histone H3 at K4 and K27, respectively, induce angiogenesis-inducing transcription [40]. Furthermore, there was also an increase in the number of genes induced by Tretinoin, which promotes angiogenesis [41,42]. This supports the idea that the blood vessels around renal cancer cells that have become PSMA-positive have enhanced angiogenic potential.

Microvesicles released from RCC cells can induce PSMA transcription. Previous studies have shown that low molecular weight fractions (10–50 kDa) of CM from the SK-RC-13 RCC, HCT-15 colon cancer, and MDA-MB-231 breast cancer cell lines induce PSMA expression in HUVECs [42].

Previous studies have shown that the CM of the PSMA-positive prostate cancer cell line LNCaP cultured on plastic-bottom dishes induces little PSMA expression in HUVECs. In contrast, in the present study, the results show that the CM from RCC cells cultured on plastic dishes and collagen I gels-induced PSMA expression in HUVECs were similar to those observed with the prostate cancer LNCaP cells. We speculated that, because the 10,000× *g* pellet fraction contains relatively large microvesicles [13,14,43,44], their release from Caki1 and ACHN cells could cause this conversion. Taken together, these data suggest that fractions containing microvesicle components released from the Caki1 and ACHN cells may transform HUVECs from PSMA-negative to PSMA-positive. 

Microvesicles can induce PSMA expression in RCC tissue that is likely endocytosed or fused to the vascular endothelial. Furthermore, the PSMA expression intensity was also clearly correlated with the extent of venous invasion. This observation suggests that the binding affinity between microvesicles and vascular endothelial cells is related to the migration of PSMA expression. However, the molecular mechanism by which microvesicles are released from RCC cells and taken up by HUVECs remains unclear. 

The signaling pathway through which microvesicles induce endothelial cell angiogenesis remains to be elucidated. However, the data from our experiments with the various CM fractions suggests that microvesicles released from renal cell carcinoma cells promoted angiogenesis in vitro. Consequently, endocytosis or the fusion of PSMA-positive membranes in normal endothelial cells may be an attractive target for the development of new anti-angiogenic agents that inhibit the transformation of normal endothelial cells into PSMA-positive tumor endothelial cells. Finally, the detection of growth factors including GDF15, HGS, and MYDGF in the 10,000× *g* pellet fraction indicates that these factors may contribute to enhancing angiogenesis. However, further investigations are required to elucidate the details of the underlying mechanisms mediating these effects.

What this study has shown is that cancer cells communicate with cells in the tumor microenvironment through microvesicles they release and convert the environment into one that is conducive to their own proliferation. PSMA-positive peritumoral blood vessels with enhanced angiogenesis supply energy to the tumor, helping it to proliferate, and enabling accelerated tumor growth. A treatment that interrupts this vicious cycle of their crosstalk must be a promising therapeutic strategy.

## 5. Conclusions

Our findings demonstrated that microvesicle components secreted by RCC cells transform vascular endothelial cells into PSMA-positive and enhance angiogenic activity. Further elucidation of the PSMA-positive process in normal vascular endothelial cells may lead to the development of novel anti-angiogenic drugs targeting the conversion of the normal vascular endothelium into tumor vessels.

## Figures and Tables

**Table 1:** List of antibodies used in this study. IHC: immunohistochemistry, IF: immunohistochemistry, WB: Western blotting.

Antibody	Species	Cat. No.	Dilution	Suppllier	Use for
CD31	Rabbit	ab28364	1/50	Abcam, Cambridge, UK	IHC
PSMA (3E6)	Mouse	M3620	1/1000	Dako, Jena, Germany	IHC
PSMA	Rabbit	12702S	1/1000	Cell Signaling Technology, Danvers, MA, USA	WB & IF
GAPDH (5A12)	Mouse	016-25523	1/6000	Wako, Osaka, Japan	WB
HRP-conjugated	Rabbit	W4011	1/2000	Promega, Madison, WI, USA	WB (secondary)
HRP-conjugated	Mouse	W4021	1/2000	Promega, Madison, WI, USA	WB (secondary)
Cy3-conjugated	Rabbit	A10520	1/500	Molecular Probes, Eugene, OR, USA	IF (secondary)
Alexa488-conjugated	Mouse	A11001	1/500	Molecular Probes, Eugene, OR, USA	IF (secondary)
Alexa488-conjugated	Rabbit	A21206	1/500	Life Technologies Corporation, Carlsbad, CA, USA	IF (secondary)
Alexa594-conjugated	Mouse	A21203	1/500	Life Technologies Corporation, Carlsbad, CA, USA	IF (secondary)

## Data Availability

The raw and processed data of spatial transcriptomics generated in this study are openly available in GEO at https://www.ncbi.nlm.nih.gov/geo/, (accessed on 21 December 2024) GEO number {GSE289717}.
